# The importance of pharmacist engagement in diagnostic stewardship

**DOI:** 10.1017/ash.2024.34

**Published:** 2024-04-16

**Authors:** Kimberly C. Claeys, Daniel J. Morgan, Melissa D. Johnson

**Affiliations:** 1 Associate Professor Infectious Diseases, University of Maryland School of Pharmacy, Baltimore, MD, USA; 2 Department of Epidemiology and Public Health, University of Maryland School of Medicine, Baltimore, MD, USA; 3 Veterans’ Affairs Maryland Healthcare System, Baltimore, MD, USA; 4 Professor in Medicine, Division of Infectious Diseases & International Health, Duke University School of Medicine, Durham, NC, USA; 5 Liaison Clinical Pharmacist, Duke Antimicrobial Stewardship Outreach Network (DASON), Duke University Medical Center, Durham, NC, USA

## Abstract

Diagnostic stewardship is increasingly recognized as a powerful tool to improve patient safety. Given the close relationship between diagnostic testing and antimicrobial misuse, antimicrobial stewardship (AMS) pharmacists should be key members of the diagnostic team. Pharmacists practicing in AMS already frequently engage with clinicians to improve the diagnostic process and have many skills needed for the implementation of diagnostic stewardship initiatives. As diagnostic stewardship becomes more broadly used, all infectious disease clinicians, including pharmacists, must collaborate to optimize patient care.

## Diagnostic stewardship, diagnostic error, and infectious diseases

Diagnostic stewardship involves modifying the ordering, processing, and/or reporting of diagnostic tests to decrease diagnostic error and improve patient quality of care.^1,2^ Diagnostic stewardship implements system-based interventions across the diagnostic process to reduce errors that occur through misdiagnosis or missed diagnosis. The modern concept of diagnostic stewardship builds off existing principles of laboratory stewardship and the National Academy of Medicine’s (NAM) focus on decreasing diagnostic error.^2,3^ Diagnostic stewardship extends beyond the diagnosis of the individual and aims to change both diagnostic and treatment paradigms to improve patient outcomes. As such, optimal diagnostic stewardship involves multiple healthcare providers along the continuum of the diagnostic process, all with unique knowledge bases and skillsets that complement the diagnostic process.^4^

Diagnostic errors are common; upwards of 1 in 20 patients in acute or ambulatory care will experience a diagnostic error, and an estimated 800,000 Americans will die or become permanently disabled annually.^3,5^ More so, infections are among the “big three” causes of diagnostic error leading to medical malpractice claims in the United States.^5^ In fact, a recent study estimated that there are more than 600,000 infection-related diagnostic errors in the United States annually from missed or delayed diagnosis alone, not considering overdiagnosis secondary to inappropriate/excessive diagnostic tests.^6^ Improving infectious disease diagnosis often focuses on advanced diagnostic testing strategies, without consideration for the need to steward these resources and prevent overdiagnosis.^2^ The growing complexity of healthcare administration, combined with the ever-increasing options for advanced diagnostic testing options, highlights the emergent need for new innovative collaborations to improve the diagnosis of infectious diseases. Pharmacists with infectious diseases/antimicrobial stewardship expertise can be leaders and key collaborators in diagnostic stewardship efforts.

## Diagnostic error in infectious diseases leads to antimicrobial misuse

Inappropriate diagnostic testing and test interpretation lead to inappropriate antimicrobial use. Half of inpatient antimicrobial use is considered inappropriate or unnecessary, often driven by diagnostic testing.^7–9^ Antimicrobial overuse leads to serious threats to both individual patients and population health. Upwards of 20% of patients on antimicrobials experience adverse drug events, and many occur in patients without clinical indications for antimicrobials.^10^ Excessive unnecessary antimicrobial exposure also leads to increased antimicrobial resistance; each additional day of broad-spectrum therapy increases the risk of infection with a multidrug-resistant organism.^11^ With rates of unnecessary antimicrobial treatment exceeding 70% in conditions such as asymptomatic bacteriuria, the need for improving diagnosis and tempering inappropriate antibiotic use through diagnostic stewardship is imperative.^12^

## Antimicrobial stewardship pharmacists and diagnostic stewardship

The NAM report on “Improving Diagnosis in Healthcare” stresses the importance of interdisciplinary teams to decrease diagnostic errors; this includes pharmacists.^3^ The Leapfrog Group also recommends the formation of multidisciplinary teams, which include pharmacists.^13^ The Centers for Disease Control and Prevention (CDC) Core Elements of Hospital Antibiotic Stewardship Programs highlight the need to incorporate diagnostic stewardship into AMS programs.^14^ With diagnostic stewardship becoming more broadly adopted, now is the time to be mindful of how we can best position these programs to have the necessary resources to improve patient care. An essential part of this discussion is determining key members of the diagnostic stewardship team and what roles these members fulfill. Antimicrobial stewardship pharmacists have established clinical relationships, skillsets suited to support diagnostic stewardship, and accessibility across healthcare systems. As dedicated diagnostic stewardship teams are formed, AMS pharmacists should not be overlooked as key members of these teams.

Antimicrobial stewardship programs implement initiatives to improve patient safety through optimizing antimicrobial use. As such, AMS pharmacists are equipped with a broad range of skills that are built on clinician engagement, behavioral economics, and implementation science. The proficiencies of AMS programs and their pharmacists can easily be leveraged to implement diagnostic stewardship initiatives. For example, pharmacists are proactive users of the electronic medical record (EMR).^15^ Pharmacists detect diagnostic errors through close review of patient records, including routine interpretation of laboratory results such as antimicrobial susceptibilities, white blood cell count, and serum creatinine. Through EMR review, a pharmacist was able to clarify the correct diagnosis and eliminate overtreatment of latent syphilis in a patient with penicillin allergy, thus preventing unnecessary hospital admission.^16^ In certain settings, pharmacists routinely order diagnostic tests as part of protocols or collaborative practice agreements. In a cross-sectional survey of pharmacists from 44 states, 71% of hospital pharmacists report they recommend cultures or laboratory tests.^17^ Additionally, AMS pharmacists can audit the use of diagnostic tests, removing duplicative unnecessary testing from order sets or assisting in targeting clinical services for education and clinical detailing.^4,18^ The skillsets needed to ensure the success of diagnostic stewardship programs are more relevant than the individual’s professional background. So, although pharmacists are not responsible for routine diagnoses, they are well equipped to influence provider behavior and implement system-wide initiatives for most common diagnoses that drive unnecessary antimicrobial use.

Strong relations with infection prevention, health information technology, and a wide array of frontline clinicians are all necessary for the implementation of effective diagnostic stewardship strategies. Within AMS program, key relationships are already in place, from the Clinical Microbiology to Chief Patient Quality and Safety Officers. Additionally, AMS pharmacists already frequently work with other healthcare providers to improve ordering and reporting of diagnostic tests for infectious diseases. For example, AMS pharmacists advocate against obtaining urine cultures in asymptomatic patients or recommend cessation of therapy in patients colonized with *Clostridioides difficile*. Given strong relationships with clinical microbiology, AMS pharmacists often serve as liaisons between the laboratory and frontline clinicians. This includes contributing to microbiology plate rounds by providing patient-specific insights.^19^ When a hospital is considering changing its antimicrobial susceptibility testing panels or adding a new molecular rapid diagnostic test (mRDT), AMS pharmacists work with health IT and clinical microbiology to optimize the impact of these tests on downstream antimicrobial use.^20^

**Figure 1.**
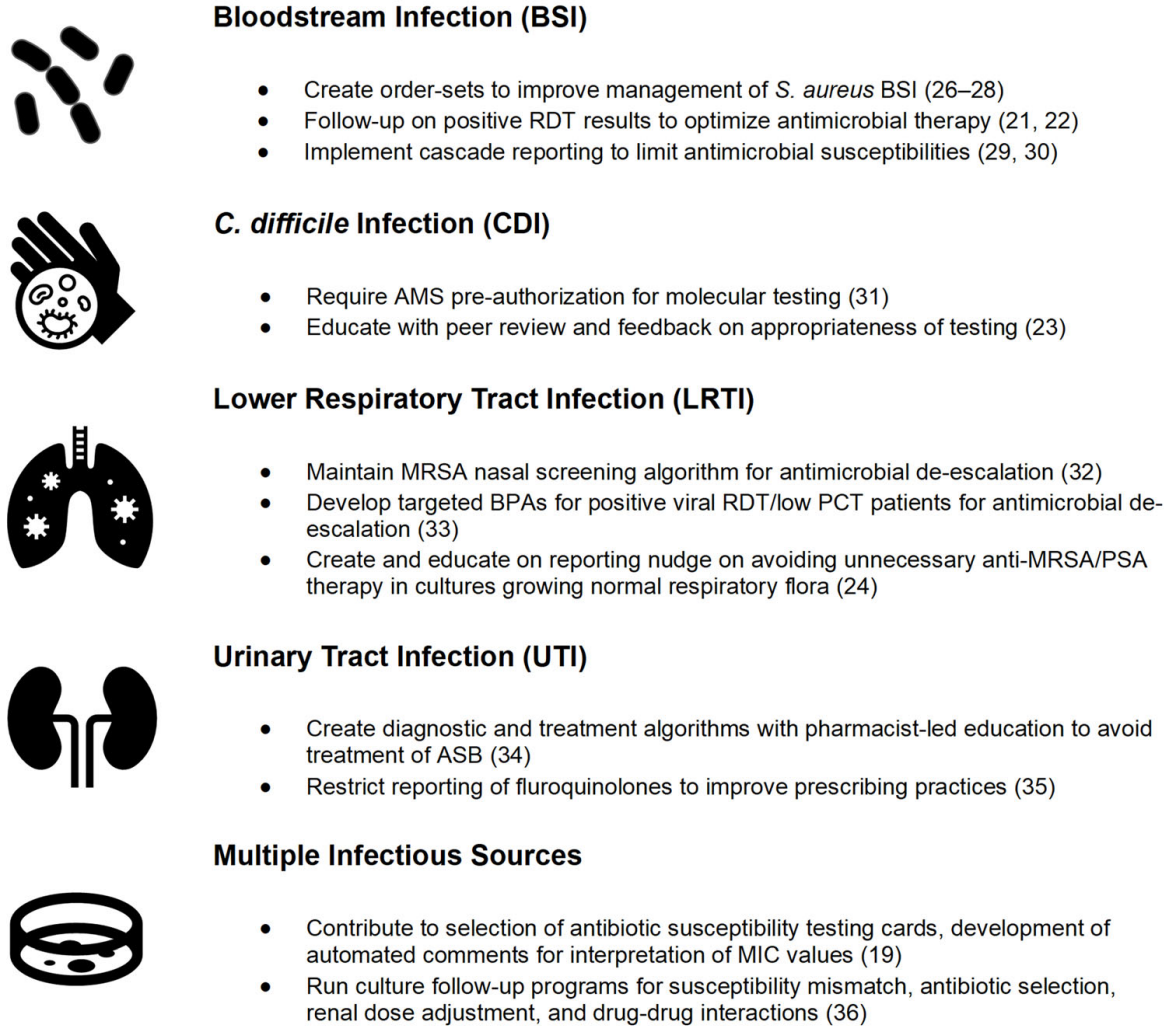
Examples of pharmacist-led diagnostic stewardship activities. *Note*: ASB, asymptomatic bacteriuria; BPA, best practice alert; BSI, bloodstream infection; CDI, *Clostridioides difficile* infection; LTRI, lower respiratory tract infection; MIC, minimum inhibitory concentration; PCT, procalcitonin; PSA, *Pseudomonas aeruginosa*; RDT, rapid diagnostic test. *Examples included in cited references.

There are several examples of successful diagnostic stewardship interventions led by pharmacists in the literature (Figure 1). These often occur at large academic medical centers through quality improvement initiatives. A commonly cited example of pharmacists’ involvement in diagnostic stewardship is the implementation of prospective review and feedback of culture or mRDT results in patients with bloodstream infections (BSI).^21,22^ A recently published AMS intervention leveraged AMS pharmacists to reduce inappropriate ordering of *C. difficile* testing, decreasing National Healthcare Safety Network Standardized Infection Ratio (SIR) and oral vancomycin days of therapy.^23^ AMS pharmacists have also decreased inappropriate antimicrobial use by improving the diagnosis of lower respiratory tract infections. For example, a pharmacist-led initiative that combined report nudging and clinical detailing significantly decreased unnecessary broad-spectrum anti-Methicillin-resistant S. aureus and anti-pseudomonal therapy in patients with respiratory cultures growing only normal flora.^24^ Pharmacists also routinely spearhead programs aimed at diagnostic safety through penicillin allergy assessment and de-labeling. De-labeling of penicillin allergies has become a focus of healthcare providers, as misdiagnosis of these allergies often leads to suboptimal care and worse clinical outcomes.^25^ Pharmacists have been leveraged to implement multidisciplinary de-labeling initiatives, with pharmacist-driven programs in place for nearly 20 years. Although many view prospective review and feedback on mRDT results for BSI as the crux of pharmacist involvement in infectious disease diagnostic stewardship, pharmacists play a pivotal role in improving diagnosis in myriad ways for numerous types of infections and patient populations. Not including pharmacists as essential members of dedicated diagnostic stewardship teams would hinder future innovative practices such as those described above.

## Conclusion

Diagnostic stewardship is growing beyond academic infection prevention and AMS programs. To effectively expand beyond these confines and continue advancing its core principles, diagnostic stewardship programs need dedicated leadership, personnel (including pharmacists), and resources. Moving forward, these programs must pay close attention to clinicians with the necessary skills to optimize outcomes and gain organizational support to improve diagnosis and patient outcomes. Antimicrobial stewardship pharmacists are a potentially untapped resource in many hospitals. The tools used in AMS programs, including behavioral and implementation sciences, patient safety, and information technology, are the same needed for diagnostic stewardship. Given the close relationship between diagnostic testing and antimicrobial use, AMS pharmacists are well suited to this work.
